# Older Adults in the United States Have Worse Cardiometabolic Health Compared to England

**DOI:** 10.1093/geronb/gbac023

**Published:** 2022-02-26

**Authors:** Benedetta Pongiglione, George B Ploubidis, Jennifer B Dowd

**Affiliations:** Centre for Research on Health and Social Care Management (CERGAS), Bocconi University, Milano, Italy; Institute of Education, University College London, London, UK; Leverhulme Centre for Demographic Science, University of Oxford, Oxford, UK

**Keywords:** Body mass index, Cardiometabolic risk, Cardiovascular disease, Obesity

## Abstract

Explanations for lagging life expectancy in the United States compared to other high-income countries have focused largely on “deaths of despair,” but attention has also shifted to the role of stalling improvements in cardiovascular disease and the obesity epidemic. Using harmonized data from the U.S. Health and Retirement Study and English Longitudinal Study of Ageing, we assess differences in self-reported and objective measures of health, among older adults in the United States and England and explore whether the differences in body mass index (BMI) documented between the United States and England explain the U.S. disadvantage. Older adults in the United States have a much higher prevalence of diabetes, low high-density lipoprotein cholesterol, and high inflammation (C-reactive protein) compared to English adults. While the distribution of BMI is shifted to the right in the United States with more people falling into extreme obesity categories, these differences do not explain the cross-country differences in measured biological risk. We conclude by considering how country differences in health may have affected the burden of coronavirus disease 2019 mortality in both countries.

Improvements in life expectancy have slowed in both England and the United States in the past decade. In England, the stall was notable in the most deprived areas of the country, especially for women (Marmot, 2020). U.S. life expectancy fell for three consecutive years starting in 2014, with increases in mortality rates seen for adults aged 25–64 years and for all race/ethnic groups (Woolf & Schoomaker, 2019). Both countries suffer from worse population health metrics compared to other European or high-income countries (Ho & Hendi, 2018; Iacobucci, 2018). While “deaths of despair” have attracted attention as a cause of lagging U.S. life expectancy (Case & Deaton, 2020), others have pointed to an important role of obesity and deaths due to cardiovascular disease (Masters et al., 2017; Mehta et al., 2020).

Recently, coronavirus disease 2019 (COVID-19) has brought comparative population health into sharp relief. Researchers have long contrasted health and health inequalities in the United States and England given the sociocultural similarities coupled with stark differences in health care provision and social protection policies. The United Kingdom and the United States have been among the countries hit most severely by the COVID-19 pandemic, both in terms of overall mortality and socioeconomic inequalities in infection and mortality rates (Iacobucci, 2020; Rose et al., 2020; Stokes et al., 2020). The reasons behind COVID-19 mortality differences within and across countries are complex and will take time to elucidate, but one potential mechanism is differences in the prevalence of underlying health conditions such as high blood pressure, obesity, and diabetes which are associated with increased risk of COVID-19 mortality. A recent meta-analysis of more than 200 studies found that among COVID-19 patients, hypertension was associated with higher severity, intensive care unit (ICU) admission, acute respiratory distress syndrome, and mortality; chronic obstructive pulmonary disease was the strongest predictor for COVID-19 severity, admission to ICU, and mortality, and patients with obesity were at a higher risk of experiencing severe symptoms of COVID-19 (Geng et al., 2021). The distribution of chronic conditions and related risk factors within and across populations is thus likely to contribute to the burden of COVID-19 mortality.

Previous studies have shown worse health in the United States compared to England, measured by chronic conditions and health-related behaviors despite their much higher levels of health care spending (Banks et al., 2006). Banks et al. (2006) compared the health status of older individuals in England and the United States in 2002 and found that older adults in the United States were generally worse off compared to their English counterparts. Differences were not solely driven by the disadvantaged end of the socioeconomic status (SES) distribution and in fact the most advantaged SES group in the United States was also less healthy compared to its English counterparts (Banks et al., 2006). Risk factors including obesity and smoking were not found to account for the U.S. disadvantage; the authors hypothesized that while the obesity epidemic hit both countries hard, the timing and intensity varied in ways that might affect the cohorts studied differently. Indeed, the prevalence of obesity rose from 7% to 23% between 1980 and 2003 in the United Kingdom compared to from 15% to 31% over the same period for the United States. The effect of long-term obesity is therefore likely to differ across countries, and its role in explaining their differences in health outcomes has not been widely explored (Wang et al., 2011).

Fifteen years later, Choi et al. (2020) updated the study by Banks et al. focusing on the Baby Boom generation (born between 1946 and 1964) who were entering the 55- to 64-year age group and are known to have worse health than earlier generations. They expanded the health indicators considered, including five self-assessed outcomes, such as functional limitation, activity of daily living (ADL), instrumental ADL (IADL), self-rated health, and depression, and used a more granular income distribution compared to the tertiles considered by Banks et al., because income gradients in health are typically steeper at lower levels of income. Overall, their findings confirmed the U.S. health disadvantage compared to England and steeper income gradient in health among Americans than English for this cohort for almost all health outcomes, with U.S.–England inequalities more pronounced among lower-income adults. Both across and within countries, the lowest-income adults in the United States had the worst outcomes for nearly all 16 measures. In the highest U.S. income group, four outcomes were worse compared to their English counterparts, and none was better. The authors suggested that more limited access to health care and social welfare programs in the United States compared with the United Kingdom may contribute to worse overall health compared with that in England, especially for lower-income U.S. adults.

Our study builds on this previous research and extends the comparison of the health of older adults in the United States and England. We examine differences across a broader age range, expand the cardiometabolic risk (CMR) factors considered, and look more closely at the explanatory role of body mass index (BMI). In particular, we test whether the differences in the full range of BMI explain the U.S. disadvantage, particularly for CMR. Levels of CMR among older Americans have remained relatively stable over the past decade but with different patterns across specific indicators. For example, levels of blood pressure, resting heart rate, and total cholesterol have decreased while others such as glycosylated hemoglobin (HbA1c), waist circumference, and pulse pressure have increased from 2006/2008 to 2014/2016 (Wu et al., 2021). The prevalence of CMR factors in the English population has been explored among the working-age population comparing birth cohorts (Jivraj et al., 2020), but is not as well documented overall. Our paper will fill the gap while comparing these harmonized measures with older adults in the United States.

Through this work, we also contribute to the early explorations of the determinants of country differences in the impact of the COVID-19 pandemic, looking at the age distribution of COVID-19 mortality and excess mortality and discuss whether these patterns may be related to prepandemic differences in health vulnerability in England and the United States. In doing so, we capitalize on results obtained from surveys of representative populations of the United States and United Kingdom to descriptively interpret national vital statistics.

## Method

### Data

Data come from two harmonized national aging studies, the Health and Retirement Study (HRS) in the United States (Sonnega et al., 2014) and the English Longitudinal Study of Ageing (ELSA) in England (Steptoe et al., 2013). Briefly, the HRS is a longitudinal panel study that surveys a representative sample of more than 37,000 individuals aged 50+ in the United States. It was established in 1992 and since then respondents have been interviewed biannually, with new cohorts added to maintain the representation of the population aged 50 and older. Since 2006, data collection has expanded to include biomarkers and genetics. ELSA is representative of the English population aged 50+ living in private households. Begun in 2002, ELSA is sampled from the Health Survey for England (HSE), a large annual cross-sectional survey on the health of the population of England. As with the HRS, participants are interviewed biannually and refreshment samples of participants aged 50+ are regularly added. Most of objective measures of health in ELSA are obtained from *nurse visits*. In ELSA Wave 8, only half of the sample underwent the nurse visit, selected to prioritize respondents across the ELSA cohorts with longitudinal nurse data. In HRS, since 2006, a random one-half of households have been preselected for the “enhanced face-to-face interview,” which includes collection of biomarkers and physical performance measures, with the other half of the sample selected for the following wave. To maximize comparability of the two studies, a data and information platform was created, the Gateway to Global Aging Data (g2aging.org), and harmonized data files are available for the data collected between 2002/2003 and 2012/2013. In this research we used data collected in 2016/2017 from the 13th HRS wave and 8th ELSA wave.

For analysis of COVID-19 mortality, we also used national statistics from the Office for National Statistics and the Centers for Disease Control and Prevention.

### Measures

Measures of health and health-related behaviors were selected based on the indicators used by Banks et al. and those added in the study of Choi et al., and further expanded to include additional indicators that capture physiological decline with age and the most prevalent chronic diseases. Our measures cover the most prevalent clusters of diseases, including a psychological distress, cardiometabolic diseases, and musculoskeletal pain, which may also interact to lower overall assessments of self-rated health (Slagboom et al., 2021). Our use of objectively measured in addition to self-reported indicators is particularly important for country comparisons (Banks et al., 2006).

#### Self-reported measures of health and health-related behaviors

We considered six measures of self-reported physician-diagnosed health. These included hypertension, diabetes, chronic lung disease, cancer, stroke, and heart attack. Next, six self-reported health indicators: self-rated general health on a 5-point scale (1 = excellent, 5 = poor); depression measured with the Center for Epidemiological Studies—Depression scale (0–8), classifying depressed those with a score equal to or higher than 3; the presence of physical disability was measured as difficulties in performing ADLs (1+ activities among dressing, walk across a room, bathing or showering, eating, getting in/out of bed, using the toilet) and IADLs (1+ activities among using a map, preparing a hot meal, shopping for groceries, making phone calls, taking medications, managing money); finally, a binary indicator for pain was also included based on the question of “are you are often troubled with pain?”

Self-reported health-related behaviors included smoking (never, current, or former smokers) and drinking, for which we define heavy drinkers as those reporting drinking 7 days per week.

In ELSA, all measures come from the main computer-assisted personal interviewing questionnaire except for drinking that was asked in the self-completion questionnaire. In HRS, all responses come from the core interview, specifically from the “Physical Health,” “Cognition,” and “Functional Limitations and Helpers” modules.

### Objective measures of health

Biomarkers include % HbA1c as measure of diabetes (≥6.5%); hypertension (systolic pressure ≥140 mm/Hg and/or diastolic pressure ≥90 mm/Hg); inflammation as measured by C-reactive protein (CRP; mg/l): high risk (≥3 mg/l), moderate risk (≥1 and <3 mg/l), and low risk (<1 mg/l). Cholesterol was measured by high-density lipoprotein (HDL) cholesterol: high (≥60 mg/dl), normal (40–59 mg/dl), low (<40 mg/dl). Note that for HDL cholesterol high levels reflect lower risk.

Clinical measurements of diabetes, hypertension, and cholesterol were also estimated correcting for self-reported drug-specific use, given that medication use was found to affect the prevalence of high risk of some biomarkers and therefore changes in overall CMR (Crimmins, 2021). Specifically, corrections were made when respondents reported taking lipid-lowering drugs (HDL cholesterol level—5%; Ki et al., 2011; Pereira et al., 2012), treatment for high blood pressure (+10 mmHg for DBP and SBP, respectively; Pereira et al., 2012; Tobin et al., 2005), and taking oral medication for type 2 diabetes (+1% in absolute terms for HbA1c; Bennett et al., 2011; Pereira et al., 2012).

We also considered measures of grip strength and walking speed as objective measures of functional status. Grip strength was measured using the Smedley dynamometer three times per hand; we used the mean score obtained in the dominant hand. Walking speed was measured only among participants aged 65+. The test was run in the main questionnaire only among participants able and willing to walk and consisted in walking 8 feet (2.5 m) at usual pace, twice. We considered the mean speed (m/s) of the two trials.

Finally, weight and height were measured in the nurse visit (height not in every nurse visit) and we estimated BMI as kg/m^2^. Based on this objective measure, we distinguished normal weight (BMI = 18–24.9), overweight (25–29), Class I obesity (30–34), Class II obesity (35–39), Class III obesity (40+).

### Analytical Strategy

Disease prevalence was estimated for each country for the total sample and by age group (50–64, 65–74, 75–84, and 85+). All proportions were estimated using the appropriate sample weights (cross-sectional and nurse-specific). Disease prevalence is first estimated adjusting for age and sex and then compared with adjustment for continuous BMI. We used a linear regression model for continuous health outcomes, logit regression for binary outcomes, and ordered logistic regression for ordinal health outcomes. All models were run both separately by country and in a pooled model with a country indicator to visualize differences across countries. To explore whether differences in chronic disease and biomarkers are consistent with different vulnerability to COVID-19 in England and United States, we examined the age distribution of COVID-19 deaths and excess mortality due to COVID-19 across the two countries. Excess mortality is calculated as the “*p*-score” (2020 deaths minus expected deaths 2015–2019)/(expected deaths 2015–2019; Aron & Muellbauer, 2020). All analyses were conducted using STATA 16.

## Results

### Health Differences Across Countries

Table 1 shows the prevalence (95% confidence interval [CI]) of each self-reported health measure in England and the United States, adjusted for age and sex. Results adjusted by BMI are included in Supplementary Table A1.

**Table 1.** Self-Reported Health Outcomes and Health-Related Behaviors in England and the United States, Ages 50+, Prevalence (95% Confidence Interval)

	England	United States
Unweighted sample *N*^a^	5,984	6,683
Hypertension	36.2 (34.6–37.9)	55.6 (54.0–57.3)
Diabetes	11.8 (10.9–12.8)	22.6 (21.3–24)
Chronic lung diseases	5.1 (4.4–5.7)	9.8 (8.8–10.8)
Cancer	5.1 (4.4–5.7)	14.4 (13.2–15.6)
Stroke	4 (3.4–4.5)	5.5 (4.7–6.2)
Heart attack	3.6 (3.0 – 4.1)	5.0 (4.3 – 5.8)
Self-rated general health		
Excellent	8.5 (7.3–9.5)	8.2 (7.2–9.1)
Very good	33.4 (31.5–35.3)	32.8 (31.2–34.4)
Good	34.5 (32.9–36.1)	34.7 (33.1–36.3)
Fair	18.2 (16.8–19.7)	18.7 (17.5–19.9)
Poor	5.4 (4.6–6.2)	5.6 (4.8–6.3)
Depression (CES-D scale ≥ 3)	19.3 (17.9–20.6)	18.7 (17.5–19.9)
ADL ≥1	15.1 (14–16.2)	13.4 (12.3–14.5)
IADL ≥1	14.9 (13.8–16)	31.2 (29.7–32.8)
Often troubled with pain	41.9 (40.2–43.6)	40 (38.4–41.6)
Ever smoked cigarettes	62.7 (61–64.4)	54.9 (53.3–56.6)
Current smoker	10.1 (8.9–11.2)	13.5 (12.3–14.7)
Heavy drinking	29.1 (27.4–30.8)	11.7 (10.6–12.7)

*Notes*: ADL = activity of daily living; CES-D scale = Center for Epidemiological Studies—Depression scale; IADL = instrumental activity of daily living.

^a^See Supplementary Figure A1 for the STROBE (Strengthening the Reporting of Observational Studies in Epidemiology) flow chart for sample selection.

In the full sample, Americans had a higher prevalence of most health conditions including hypertension (55.6% [95% CI: 54.0–57.3] in the United States vs 36.2% [34.6–37.9] in England) and diabetes (22.6% [21.3–24.0] in the United States vs 11.8% [10.9–12.8] in England), as well as in less common diseases, such as chronic lung disease and cancer, for which the U.S. prevalence was almost twice that of England. Heart attack and stroke were also more frequently reported in the United States. Prevalence of poor self-reported health, pain, and depression were not statistically different between the two countries.

Table 2 shows results for the biomarker sample, including comparisons of self-reported hypertension and diabetes with biomarker-assessed values. As seen in Table 1, hypertension was much more frequently reported by Americans; in the subsample who undertook the nurse visit, the prevalence of self-reported hypertension was similar, but the prevalence of objectively measured hypertension was much lower overall and similar between countries (28.3% [26.1–30.5] in England and 26.9% [25.2–28.6] in the United States). For diabetes, the clinically measured prevalence was lower than the self-reported prevalence, but the English advantage was observed for both indicators, as well as when correcting for medication use. High-risk levels of CRP were much higher in the United States (34.5% [32.7–36.3] in the United States vs 25.3% [23.3–27.4] in England), and HDL cholesterol levels were higher (healthier values) in England compared to the United States (50.6% [48.2–53] vs 41.3% [39.3–43.2]).

**Table 2:** Biomarker Outcomes, England and the United States, Ages 50+, Prevalence/Mean (95% Confidence Interval)

	England	United States
Unweighted sample *N*^a^	2,203	4,596
Diabetes, HbA1c ≥6.5		
Prevalence, self-report	10.2 (8.7–11.6)	21.3 (19.8–22.7)
Prevalence, clinical report	8.8 (7.4–10.3)	13.1 (11.9–14.3)
Prevalence, clinical report corrected for medications	10.3 (8.8–11.8)	18.8 (17.4–20.3)
Hypertension, systolic blood pressure ≥140 mmHg, diastolic ≥90 mmHg, or taking medication, %		
Prevalence, self-report	35.3 (32.9–37.7)	55.9 (54–57.8)
Prevalence, clinical report	28.3 (26.1–30.5)	26.9 (25.2–28.6)
Prevalence, clinical report adjusted for medications	36.3 (34–38.7)	40 (38.1–41.8)
C-reactive protein, mg/l		
Low risk, ≤1, %	37.1 (34.7–39.4)	27.5 (25.7–29.2)
Moderate risk, 1–3, %	37.6 (35.8–39.5)	38 (36.2–39.9)
High risk, ≥3, %	25.3 (23.3–27.4)	34.5 (32.7–36.3)
Mean	3.1 (2.7–3.4)	3.6 (3.4–3.7)
HDL cholesterol, mg/dl		
Low, 40, %	11.2 (9.9–12.4)	15.5 (14.2–16.8)
Normal, 40–60, %	38.2 (36.3–40.2)	43.2 (41.3–45.1)
High, 60, %	50.6 (48.2–53)	41.3 (39.3–43.2)
Adjusted for medications: Low, 40, %	12.8 (11.4–14.2)	19.3 (17.8–20.7)
Adjusted for medications: Normal, 40–60, %	38.5 (36.6–40.5)	43.9 (42–45.8)
Adjusted for medications: High, 60, %	48.7 (46.2–51.2)	36.8 (35–38.7)
Mean	62.3 (61.3–63.2)	58.3 (57.6–59)
Mean adjusted for medications	61.1 (60.2–62)	56.2 (55.6–56.9)
BMI		
Normal weight	28 (25.9–30.1)	19.2 (17.7–20.8)
Overweight	38.9 (36.9–40.9)	36.1 (34.3–37.9)
Class I obesity	20.9 (19.3–22.5)	26.3 (24.5–28)
Class II obesity	8.5 (7.4–9.5)	12.6 (11.3–13.8)
Class III obesity	3.7 (3–4.3)	5.8 (4.9–6.7)
Mean	28.4 (28.1–28.7)	30 (29.8–30.2)
Grip strength (kg), mean	30.5 (30.2–30.9)	30.2 (29.9–30.5)
Walking speed (m/s)^b^, mean	3.3 (3.2–3.4)	3.7 (3.7–3.8)

*Notes*: BMI = body mass index; Class I obesity: BMI 30–35; Class II obesity: BMI 35–39; Class III obesity: BMI ≥40; HbA1c = glycosylated hemoglobin; HDL= high-density lipoprotein.

^a^See Supplementary Figure A1 for the STROBE (Strengthening the Reporting of Observational Studies in Epidemiology) flow chart for sample selection.

^b^Unweighted sample size (aged 65+) *N* = 1,441 in England, *N* = 2,345 in the United States.

**Figure 1.**
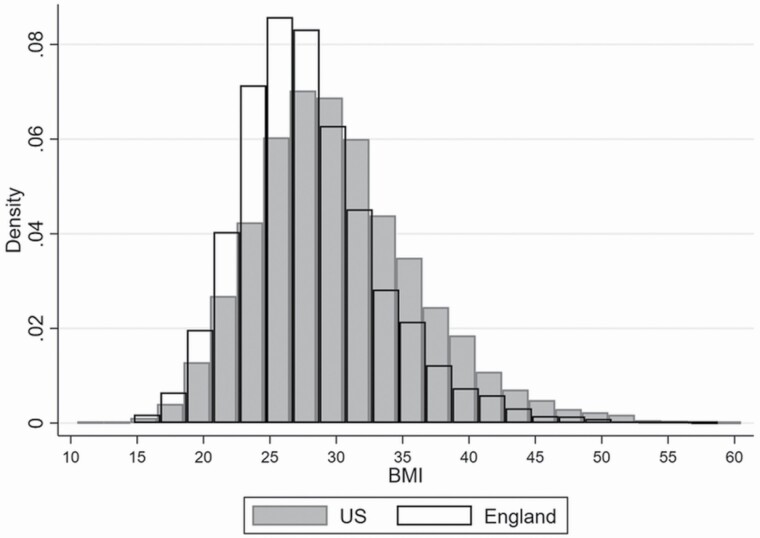
Distribution of body mass index (BMI) in England and the United States, ages 50+.

Figure 1 examines the distribution of BMI across the two countries (Supplementary Figure A2 shows it by age group). The distribution in the United States is flatter and shifted to the right compared to England, with more density in the highest obesity classes most associated with chronic disease. For both countries, the mean and median BMI are above the threshold for overweight (BMI ≥25). In the United States the mean BMI = 30.0, median BMI = 29.4; in England mean BMI = 28.4, median BMI = 27.3. Table 2 also shows the proportions of the ELSA and HRS population in each detailed BMI category. Notably, the United States has an 18.4% prevalence of Class II or III obesity (BMI ≥35) compared to 12.1% in England.

Many cardiometabolic biomarkers have strong associations with body weight, especially markers of inflammation such as CRP (McDade et al., 2021). Despite more extreme levels of obesity in the United States, country comparisons remained substantially unchanged when adjusting for continuous BMI (results in Supplementary Tables A1 and A2), with few exceptions such that the English advantage in a few outcomes slightly reduced when differences in BMI were accounted for. These include a smaller U.S.–England difference in the prevalence of self-reported hypertension (19.4% higher in the United States based on the sex–age-adjusted estimates and 17.6% higher in the United States based on the sex–age–BMI-adjusted estimates), self-reported diabetes (10.8% gap when not adjusting for BMI vs 9.5% gap adjusting for BMI), and high CRP levels (9.2% gap when not adjusting for BMI vs 5.9% gap adjusting for BMI). The direction of changes indicates that adjusting for current BMI accounts for a small amount of the England–U.S. health gap, but most of the differences persist and require further explanation. Indeed, even for the conditions just described, the reduction of the U.S.–England gap was modest and the CIs of the estimates obtained adjusting and not adjusting for BMI overlapped.

**Figure 2.**
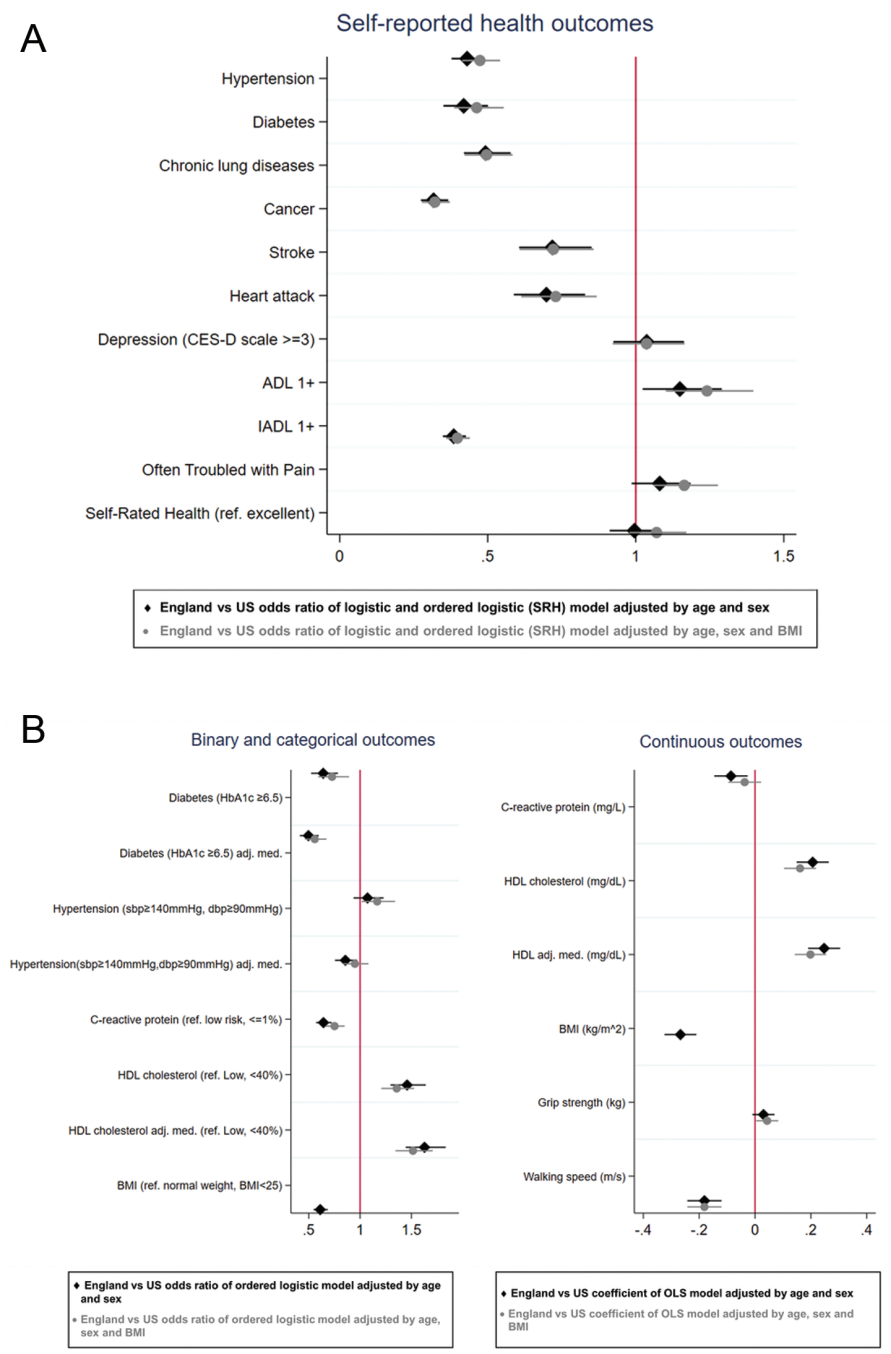
Country differences in health outcomes. (A) Self-reported health outcomes. (B) Objective measures of health (biomarkers). *Note*: Categories of HDL cholesterol are coded “Low” <40%, “normal” 40–60%, “high” 60%, higher HDL category corresponds to better health status, similarly for continuous variable higher values correspond to better health status. For walking speed higher values correspond to poorer health status. For grip strength higher values correspond to better health status. ADL = activity of daily living; CES-D = Center for Epidemiological Studies—Depression; HDL = high-density lipoprotein; IADL = instrumental activity of daily living.

Figure 2 summarizes these country differences in outcomes by reporting the country odds ratio (England vs United States) or beta coefficient, respectively, for binary/categorical health outcomes and continuous health outcomes, obtained from the model adjusted for age and sex (black marks; from which estimates presented in Tables 1 and 2 are taken), and for the model that also controls for BMI (gray marks; from which estimates presented in Supplementary Tables A1 and A2 are taken). Figure 2A shows results for self-reported measures and Figure 2B for biomarkers, separately for binary/categorical (left column) and continuous indicators (right column). Again we see the health advantage for England for almost all health outcomes, with the advantage almost unchanged adjusting for BMI. In Supplementary Figure A3, we also include the results obtained from a model adjusted only for sex and BMI, and estimates do not change for any outcome.

Finally, we calculated the prevalence of self-reported and objectively measured health indicators separately by age groups and adjusting for sex and continuous age to see if patterns of health differences across countries are consistent across age. Results are available in Supplementary Tables A3 and A4. Similarly, we reestimated the models shown in Figure 2 separately for those aged under and over 75 (Supplementary Figure A4). As expected, the prevalence of chronic health conditions increased with age, with a few exceptions including diabetes (self-reported and clinical), depression, and self-reported pain, and high CRP. Overweight and Class I obesity were also more prevalent among younger adults, with Class II and III obesity higher in those 50–64 and 65–74 compared to those aged 85+, both in the United States and England. Country differences remained relatively stable across age groups, with no strong indication that the U.S. disadvantage is larger in the middle-aged cohorts subject to recent attention in mortality analyses, except only for self-reported hypertension and high level of CRP, for which the U.S. disadvantage was larger among those aged 50–64 and 65–74; also the prevalence of Class I and II obesity was higher in the United States compared to England particularly among these age groups. Likewise, Supplementary Figure A4 indicates that the U.S. disadvantage is slightly more predominant among individuals under 75, while among those over 75 there is an English disadvantage for depression, ADLs, pain, and self-rated general health (SRH), which is also identified in Supplementary Tables A3 and A4 which compare more refined age groups.

### Disparities in COVID-19 Outcomes Between Countries

**Figure 3.**
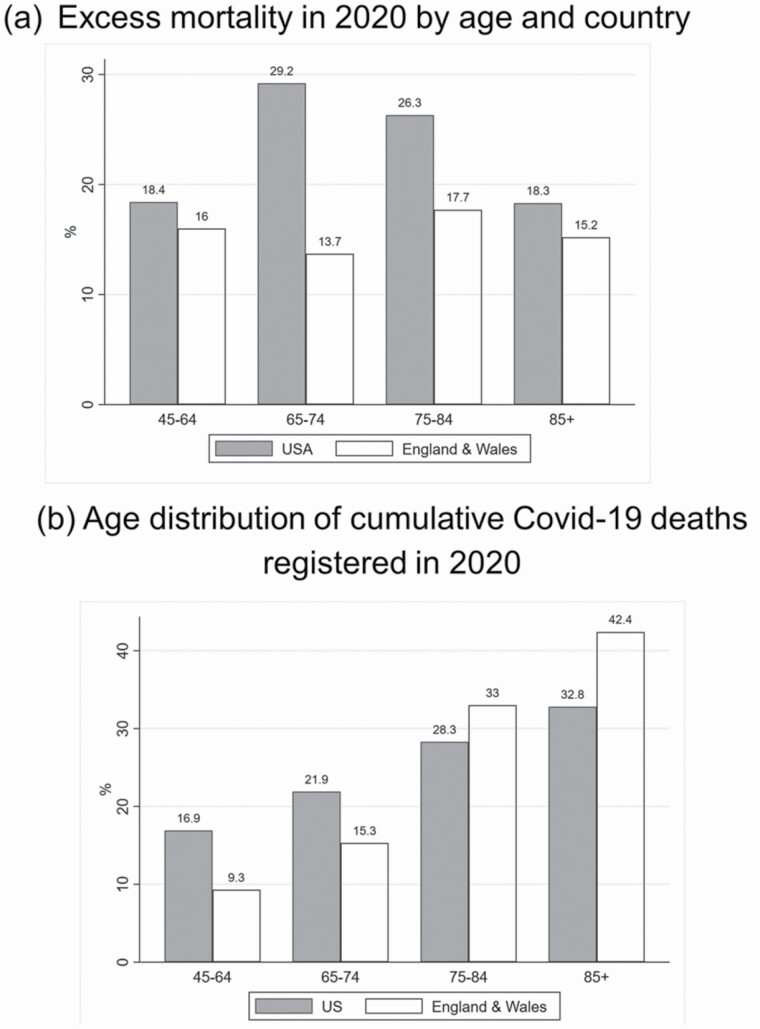
Cumulative excess mortality and official coronavirus disease 2019 (COVID-19) mortality by age group and country, March–December 2020. (a) Excess mortality in 2020 by age and country (b) Age distribution of cumulative Covid-19 deaths registered in 2020.

Next we compare differences in COVID-19 mortality across the countries as a first exploration of the hypothesis that underlying differences in the prevalence of underlying conditions contributes to country differences in COVID-19 mortality. Obesity and diabetes have both been identified as risk factors for severe COVID-19 disease and death (Zhou et al., 2021), and we found that the prevalence of both was higher among the U.S. older adults than English and across all age groups, particularly among the younger old adults. Figure 3 reports excess all-cause mortality compared to the previous 5 years using the *p*-score by age group (Figure 3A), along with the age distribution of cumulative official COVID-19 deaths (Figure 3B). Figure 3A shows much higher excess mortality in the United States compared to England and Wales, particularly among those aged 65–84, for which excess deaths are almost 30% above expected levels in the United States versus about 15% in England and Wales. Even though absolute numbers of deaths were higher at older ages, the relative increase in expected mortality was also high for the “younger” older-age groups, particularly those 65–74 in the United States. Figure 3B shows the age distribution of confirmed COVID-19 deaths occurred in 2020 in each country. Notably, the age distribution of deaths is shifted younger in the United States compared to England and Wales. While 75.4% of deaths in England & Wales occurred at age 75 or over, the corresponding percentage in the United States was only 61.1%.

In Supplementary Figure A5, the proportions of official COVID-19 deaths out of total deaths across all age groups in the two countries are also shown, and are very similar in England and the United States, suggesting a likely under-reporting of COVID-19 deaths in the United States, given the much higher excess mortality.

## Discussion

The United States and England are both falling behind European peers in life expectancy (Ho & Hendi, 2018) and have suffered relatively high COVID-19 mortality. Our study explored whether health differences among older adults in England and the United States observed in the early 2000s remain in more recent years and for a broader age range, and whether there is evidence that middle-aged cohorts in the United States are doing worse as reflected in recent age-specific mortality trends. We found evidence that the U.S. American disadvantage observed by Banks et al. persists 15 years later across a wide range of self-reported and objectively measured health indicators, and for all adults aged 50+. We also observed some striking increase in prevalence that occurred in both countries. Looking at adults aged 50–64, hence comparable with the population studied by Banks et al., the prevalence of diabetes, both self-reported and objectively measured, was remarkably higher in 2016 (our estimates [Supplementary Table A3]: 8.6% and 19.2% in England and United States, respectively) compared to 2002 in both the United States and England (Banks et al.: 6.1% in England and 12.5% in the United States). Our estimates are also in line with those of Choi et al. that studied the same time period (8.9% and 19% in England and United States, respectively). We also assessed the U.S.–England gap among individuals older than 65, and found a U.S. disadvantage in diabetes across all age groups (aged 65–74: 13.6% in England vs 26.7% in the United States; aged 75–84: 16.3% in England vs 28.5% in the United States; aged 85+: 13.2% in England and 18.7% in the United States). Similarly, the proportion of obese individuals has increased considerably in both countries, with the U.S.–England gap persisting over time (Banks et al.: 23.0% in England and 31.1% in the United States; ours estimates for those aged 50–64: 35.7% and 49.1%). On the other hand, other CMR remained stable or even declined compared to estimates from 2002, with different trajectories in the United States and England. This is the case of hypertension (33.8% in England and 42.4% in the United States in 2002 according to Banks et al. vs 27.7% in England and 45.3% in the United States based on our estimates for those aged 50–64) and HDL cholesterol improved (mean HDL = 59 in England and 52 in the United States according to Banks et al. vs 62.1 in England and 59.2 in the United States based on our estimates for those aged 50–64). Similar trajectories were observed for the older U.S. Americans in the longitudinal study of Wu et al. (2021).

The distribution of BMI among older U.S. adults is shifted to the right, with 18.4% of the sample falling into severe and morbid obesity categories compared to 12.2% in England. While Banks et al. adjusted for categorical BMI in their original analysis of adults aged 55–64, they defined obesity only as BMI >30, obscuring the potential role of differences in extreme obesity above this level. Since diabetes and inflammation are strongly correlated with body fat (Kahn et al., 2006; McDade et al., 2021), these differences in extreme obesity are a potential mechanism for explaining differences in biomarkers across countries. Despite adjusting for the full range of continuous BMI in our models, we found that current BMI explained very little of the cross-country differences in cardiometabolic measures. Our results suggest that adults aged 50 and older in the United States are metabolically less healthy than their English counterpoints, but this is not well explained by current BMI. Our findings are consistent Martinson et al. (2011), who found U.S. disadvantages in biomarkers of health risk including CRP and HDL cholesterol using 1999–2006 data from the HSE and U.S. National Health and Nutrition Examination Survey for a wide age range (Martinson et al., 2011). These differences were also only minimally attenuated adjusting for BMI. In this volume, Martinson et al. (2022) find a U.S. disadvantage in obesity prevalence in Millennials and Gen X at ages 30–34, but patterns for specific biological risk factors vary by gender and cohort and do not show a consistent U.S. disadvantage. Even though our results are in line with previous research, it is surprising that more refined adjustment BMI only explain very modestly U.S./England differences in CMR, given the BMI compositional differences shown in Figure 1. We explored the BMI distribution across age groups (Supplementary Figure A2), and consistent with results for categorical BMI shown in Table 2, the U.S. shift in the distribution mostly pertains to individuals aged 50–64 and 65–75. Hence, the association between age and health is reasonably confounded by BMI due to lower weight among older adults; but at the same time, their lower weight may be due to illness. As a sensitivity analysis, we run the models shown in Figure 2 controlling only for sex and BMI and results do not change (Supplementary Figure A3). We also stratified the sample by age separating those under and over 75 (Supplementary Figure A4), and estimated for each outcome country differences adjusting and not adjusting for BMI. While the two model specifications gave very similar results, hence suggesting BMI does not explain much of country’s differences in either age group, interesting differences emerged comparing results of those under and over 75. For those aged 75+, we observe an English disadvantage for depression, ADLs, pain, and poor SRH not observed among those under 75, while for those under 75 we observe a U.S. disadvantage in clinical diabetes and CRP not observed among those aged 75+. Suggesting that country differences for some health outcomes are not consistent across age groups, at the U.S. disadvantage among the youngest adults and at the English disadvantage among the older adults, matching the U.S./England BMI distribution across age groups and age distribution of COVID-19 deaths registered in 2020.

While weight histories are not currently available in these surveys, future work would benefit from testing whether duration of obesity or maximum BMI can explain these differences, which have been shown more predictive of mortality than current BMI in older adults (Norris et al., 2020; Stokes & Preston, 2016). In the absence of explanations from known risk factors such as BMI, smoking, and heavy drinking, the mechanisms underlying these sizeable differences in biological risk remain a puzzle. Previous work by Banks et al. (2006) and recently updated by Choi et al. (2020) comparing the HRS and ELSA found larger income and education inequalities in most health outcomes in the United States. Future work should move to incorporate such differences in life course social and environmental exposures known to be associated with these biological risk factors like early childhood adversity and other sources of psychosocial stress.

The U.S. disadvantage in measured biological risk and self-reported health outcomes is consistent with observed differences in life expectancy across the two countries. In the United States, life expectancy at birth in 2019 was 76.3 for men and 81.4 for women compared to 79.9 for men and 83.6 for women in England (Kochanek et al., 2020; Morgan & Rozée, 2020). While there has been significant attention to the role of “deaths of despair” in explaining rising middle-aged mortality in the United States, our results show no country difference in two measures of distress—self-reported pain and depression; and this held true also when we disentangled results by age groups, with the exception of a significantly higher proportion of English aged 85+ reporting pain compared to their U.S. counterparts. Our evidence on higher CMR in Americans is consistent with the work of Mehta et al. (2020) who found that cardiovascular disease mortality has contributed more to American stagnation in life expectancy than deaths of despair. These large differences in observed CMR deserve further attention for potentially explaining current and future trends in life expectancy.

Both the United States and the United Kingdom have been among the worst hit countries by the COVID-19 pandemic as measured by per capita mortality. While deaths in the United Kingdom were strongly concentrated at older ages, the United States saw more excess deaths and more relative deaths in “younger” old age groups (Aburto et al., 2022). In this special volume, Masters et al. (2022) find that the losses in life expectancy in the United States due to COVID-19 due to deaths at younger ages mirror the age-specific contributions of U.S. life expectancy disadvantage for many years prior. There are several potential explanations for this vulnerability to COVID-19 at younger ages in United States, including differences in social protection for essential workers and race/ethnic differences in infection and mortality. While much work remains to be done to understand these mechanisms, the greater prepandemic biological vulnerability of Americans aged 50+ compared to English, especially for conditions such as diabetes linked to more severe COVID-19, may be one reason for this shift of mortality to younger ages and greater overall excess mortality. Both countries experienced higher relative risks of infection and mortality among disadvantaged groups and race/ethnic minorities, but whether these COVID-19 disparities were more pronounced in the United States is not yet known. Looking ahead, the long-term health effects of COVID-19 and long COVID will likely make a lasting imprint on the population health of both countries, including health and disability at older ages.

## Supplementary Material

gbac023_suppl_Supplementary_Appendix

## References

[CIT0001] Aburto, J. M., Schöley, J., Kashnitsky, I., Zhang, L., Rahal, C., Missov, T. I., Mills, M. C., Dowd, J. B., Kashyap, R. (2022). Quantifying impacts of the COVID-19 pandemic through life-expectancy losses: A population-level study of 29 countries. International Journal of Epidemiology, 51(1), 63–74. doi:10.1093/ije/dyab207 34564730 PMC8500096

[CIT0002] Aron, J., & Muellbauer, J. (2020). Measuring excess mortality: The case of England during the Covid-19 pandemic. Institute for New Economic Thinking, Oxford Working Paper, 11.

[CIT0003] Banks, J., Marmot, M., Oldfield, Z., & Smith, J. P. (2006). Disease and disadvantage in the United States and in England. Journal of American Medical Association, 295(17), 2037–2045. doi:10.1001/jama.295.17.2037 16670412

[CIT0004] Bennett, W. L., Maruthur, N. M., Singh, S., Segal, J. B., Wilson, L. M., Chatterjee, R., Marinopoulos, S. S., Puhan, M. A., Ranasinghe, P., Block, L., Nicholson, W. K., Hutfless, S., Bass, E. B., & Bolen, S. (2011). Comparative effectiveness and safety of medications for type 2 diabetes: An update including new drugs and 2-drug combinations. Annals of Internal Medicine, 154(9), 602–613. doi:10.7326/0003-4819-154-9-201105030-00336 21403054 PMC3733115

[CIT0032] Case, A., & Deaton, A. (2020). Deaths of despair and the future of capitalism. Princeton University Press.

[CIT0005] Choi, H., Steptoe, A., Heisler, M., Clarke, P., Schoeni, R. F., Jivraj, S., Cho, T. C., & Langa, K. M. (2020). Comparison of health outcomes among high- and low-income adults aged 55 to 64 years in the US vs England. JAMA Internal Medicine, 180(9), 1185–1193. doi:10.1001/jamainternmed.2020.2802 32897385 PMC7358980

[CIT0006] Crimmins, E. M . (2021). Recent trends and increasing differences in life expectancy present opportunities for multidisciplinary research on aging. Nature Aging, 1(1), 12–13. doi:10.1038/s43587-020-00016-0 34355199 PMC8336715

[CIT0007] Geng, J., Yu, X., Bao, H., Feng, Z., Yuan, X., Zhang, J., Chen, X., Chen, Y., Li, C., & Yu, H. (2021). Chronic diseases as a predictor for severity and mortality of COVID-19: A systematic review with cumulative meta-analysis. Frontiers in Medicine, 8, 588013. doi:10.3389/fmed.2021.588013 34540855 PMC8440884

[CIT0008] Ho, J. Y., & Hendi, A. S. (2018). Recent trends in life expectancy across high income countries: Retrospective observational study. BMJ (Clinical Research Ed.), 362, k2562. doi:10.1136/bmj.k2562 PMC6092679 30111634

[CIT0033] Iacobucci, G . (2018). UK women living shorter lives than most European counterparts. BMJ, 362, k3871. doi:10.1136/bmj.k3871 30206179

[CIT0009] Iacobucci, G . (2020). Covid-19: Excess deaths vary widely across England and Wales, show data. BMJ (Clinical Research Ed.), 371, m4500. doi:10.1136/bmj.m4500 33208321

[CIT0010] Jivraj, S., Goodman, A., Pongiglione, B., & Ploubidis, G. B. (2020). Living longer but not necessarily healthier: The joint progress of health and mortality in the working-age population of England. Population Studies, 74(3), 399–414. doi:10.1080/00324728.2020.1767297 32659174

[CIT0011] Kahn, S. E., Hull, R. L., & Utzschneider, K. M. (2006). Mechanisms linking obesity to insulin resistance and type 2 diabetes. Nature, 444(7121), 840–846. doi:10.1038/nature05482 17167471

[CIT0012] Ki, M., Pouliou, T., Li, L., & Power, C. (2011). Physical (in)activity over 20 y in adulthood: Associations with adult lipid levels in the 1958 British birth cohort. Atherosclerosis, 219(1), 361–367. doi:10.1016/j.atherosclerosis.2011.07.109 21855876

[CIT0013] Kochanek, K. D., Xu, J., & Arias, E. (2020). Mortality in the United States, 2019. NCHS Data Brief, 395, 1–8. www.cdc.gov/nchs/products/databriefs/db395.htm 33395387

[CIT0034] Marmot, M . (2020). Health equity in England: The Marmot review 10 years on. BMJ, 368, m693. 32094110 10.1136/bmj.m693

[CIT0014] Martinson, M. L., Teitler, J. O., & Reichman, N. E. (2011). Health across the life span in the United States and England. American Journal of Epidemiology, 173(8), 858–865. doi:10.1093/aje/kwq325 21389038 PMC3105255

[CIT0035] Martinson, M, L., Lapham, J., Ercin-Swearinger, H., Teitler, J. O., & Reichman, N. E. (2022) Generational shifts in young adult cardiovascular health? Millennials and Generation X in the United States and England. The Journals of Gerontology, Series B: Psychological Sciences and Social Sciences, 77(S2), S177–S188. doi:10.1093/geronb/gbac036 PMC9154229 35195713

[CIT0037] Masters, R. K., Tilstra, A. M., & Simon, D. H. (2017). Explaining recent mortality trends among younger and middle-aged White Americans. International Journal of Epidemiology, 47(1), 81–88. 10.1093/ije/dyx127 PMC6658718 29040539

[CIT0038] Masters, R. K., Woolf, S. H., & Aron, L. Y. (2022). Age-specific mortality during the 2020 COVID-19 pandemic and life expectancy changes in the United States and peer countries, 1980–2020. The Journals of Gerontology, Series B: Psychological Sciences and Social Sciences, 77(S2), S127–S137. doi:10.1093/geronb/gbac028 PMC9154231 35191480

[CIT0017] McDade, T. W., Meyer, J. M., Koning, S. M., & Harris, K. M. (2021). Body mass and the epidemic of chronic inflammation in early mid-adulthood. Social Science & Medicine, 281, 114059. doi:10.1016/j.socscimed.2021.114059 34091232 PMC8259331

[CIT0018] Mehta, N. K., Abrams, L. R., & Myrskylä, M. (2020). US life expectancy stalls due to cardiovascular disease, not drug deaths. Proceedings of the National Academy of Sciences of the United States of America, 117(13), 6998–7000. doi:10.1073/pnas.1920391117 32179670 PMC7132127

[CIT0019] Morgan, E., & Rozée, S. (2020). National life tables–life expectancy in the UK: 2017 to 2019. Office of National Statistics.

[CIT0020] Norris, T., Cole, T. J., Bann, D., Hamer, M., Hardy, R., Li, L., Ong, K. K., Ploubidis, G. B., Viner, R., & Johnson, W. (2020). Duration of obesity exposure between ages 10 and 40 years and its relationship with cardiometabolic disease risk factors: A cohort study. PLoS Medicine, 17(12), e1003387. doi:10.1371/journal.pmed.1003387 33290405 PMC7723271

[CIT0021] Pereira, S. M. P., Ki, M., & Power, C. (2012). Sedentary behaviour and biomarkers for cardiovascular disease and diabetes in mid-life: The role of television-viewing and sitting at work. PLoS One, 7(2), e31132. doi:10.1371/journal.pone.0031132 22347441 PMC3276501

[CIT0022] Rose, T. C., Mason, K., Pennington, A., McHale, P., Taylor-Robinson, D. C., & Barr, B. (2020). Inequalities in COVID-19 mortality related to ethnicity and socioeconomic deprivation. medRxiv.

[CIT0023] Slagboom, M. N., Reis, R., Tsai, A. C., Büchner, F. L., van Dijk, D. J. A., & Crone, M. R. (2021). Psychological distress, cardiometabolic diseases and musculoskeletal pain: A cross-sectional, population-based study of syndemic ill health in a Dutch fishing village. Journal of Global Health, 11, 04029. doi:10.7189/jogh.11.04029 33959260 PMC8068410

[CIT0024] Sonnega, A., Faul, J. D., Ofstedal, M. B., Langa, K. M., Phillips, J. W., & Weir, D. R. (2014). Cohort profile: The Health and Retirement Study (HRS). International Journal of Epidemiology, 43(2), 576–585. doi:10.1093/ije/dyu067 24671021 PMC3997380

[CIT0025] Steptoe, A., Breeze, E., Banks, J., & Nazroo, J. (2013). Cohort profile: The English Longitudinal Study of Ageing. International Journal of Epidemiology, 42(6), 1640–1648. doi:10.1093/ije/dys168 23143611 PMC3900867

[CIT0026] Stokes, A. C., Lundberg, D .J., Elo, I. T., Hempstead, K., Bor, J., & Preston, S. H. (2021). Assessing the impact of the Covid-19 pandemic on US mortality: A county-level analysis. medRxiv. doi: 10.1101/2020.08.31.20184036 PMC8136644 34014945

[CIT0027] Stokes, A., & Preston, S. H. (2016). Revealing the burden of obesity using weight histories. Proceedings of the National Academy of Sciences of the United States of America, 113(3), 572–577. doi:10.1073/pnas.1515472113 26729881 PMC4725464

[CIT0028] Tobin, M. D., Sheehan, N. A., Scurrah, K. J., & Burton, P. R. (2005). Adjusting for treatment effects in studies of quantitative traits: Antihypertensive therapy and systolic blood pressure. Statistics in Medicine, 24(19), 2911–2935. doi:10.1002/sim.2165 16152135

[CIT0029] Wang, Y. C., McPherson, K., Marsh, T., Gortmaker, S. L., & Brown, M. (2011). Health and economic burden of the projected obesity trends in the USA and the UK. Lancet (London, England), 378(9793), 815–825. doi:10.1016/S0140-6736(11)60814-3 21872750

[CIT0036] Woolf, S. H., & Schoomaker H. (2019) Life expectancy and mortality rates in the United States, 1959–2017. JAMA, 322(20), 1996–2016. doi:10.1001/jama.2019.16932 31769830 PMC7146991

[CIT0030] Wu, Q., Ailshire, J. A., Kim, J. K., & Crimmins, E. M. (2021). Cardiometabolic risk trajectory among older Americans: Findings from the Health and Retirement Study. The Journals of Gerontology, Series A: Biological Sciences and Medical Sciences, 76(12), 2265–2274. doi:10.1093/gerona/glab205 34252185 PMC8599082

[CIT0031] Zhou, Y., Chi, J., Lv, W., & Wang, Y. (2021). Obesity and diabetes as high-risk factors for severe coronavirus disease 2019 (Covid-19). Diabetes/Metabolism Research and Reviews, 37(2), e3377. doi:10.1002/dmrr.3377 32588943 PMC7361201

