# Supplementary material for: Older Adults in the United States Have Worse Cardiometabolic Health Compared to England
Source: J Gerontol B Psychol Sci Soc Sci. 2022 Feb 26;77(Suppl 2):S167–76. doi: 10.1093/geronb/gbac023 (PMC9154237; doi:10.1093/geronb/gbac023)

**Appendix**

*Sample selection*

Figure A1 illustrates the sample selection in the two surveys. Two separate samples were considered with respect to the type of outcome, self-reports and objective measures. The much lower size of biomarkers depends on the fact that in both ELSA and HRS not all respondents undertook the nurse visit. While in HRS the sample was random, in ELSA it was a selective sample. We used ad hoc weights both in ELSA and HRS to restore sample representativeness.

**Figure A1.** HRS and ELSA sample selection

year 2016/17

(N=20,912)

Excluded (n= 771)

- Aged <50 years (n= 771)

Analysis of self-reported measures

(N=6,683)

Considered for the analysis (n=20,139)

Analysis of objective health measures

(N=4,605)

- Excluded from nurse visit (N=10,565)

Uncompleted records on SEP and self-reported health outcomes (N=13,456)


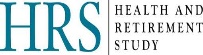


year 2016/17

(N=8,445)

Excluded (n= 85)

- Aged <50 years (n= 85)

Analysis of self-reported measures

(N=5,984)

Considered for the analysis (n=8,360)

Analysis of objective health measures

(N=2,111)

- Excluded from nurse visit (N=4,838)

Uncompleted records on SEP and self-reported health outcomes (N=2,376)


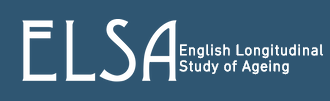


**Table A1.** Self-reported health outcomes and health-related behaviors in England and the United States, ages 50+, prevalence (95% confidence interval) adjusted for age, sex and BMI.

|  | **England** | **United States** |
| --- | --- | --- |
| Unweighted-sample N | 5,631 | 6,683 |
| Hypertension | 38.4 (36.6 - 40.2) | 56 (54.3 - 57.7) |
| Diabetes | 12.1 (11.1 - 13.1) | 21.6 (20.2 - 22.9) |
| Chronic lung diseases | 5 (4.3 - 5.7) | 9.6 (8.6 - 10.5) |
| Cancer | 5.1 (4.4 - 5.7) | 14.3 (13.1 - 15.4) |
| Stroke | 4 (3.4 - 4.5) | 5.4 (4.7 - 6.1) |
| Heart attack | 4.7 (3.9 - 5.5) | 1.8 (1.4 - 2.3) |
| All heart diseases | 12.6 (11.6 - 13.7) | 21.7 (20.3 - 23.1) |
| Self-rated general health: Excellent | 7.8 (6.8 - 8.6) | 8.3 (7.3 - 9.1) |
| Very good | 32.2 (30.5 - 33.9) | 33.4 (31.8 - 34.9) |
| Good | 35.7 (34.1 - 37.2) | 35.3 (33.7 - 36.8) |
| Fair | 19.2 (17.9 - 20.6) | 18.3 (17.1 - 19.5) |
| Poor | 5.2 (4.4 - 5.9) | 4.8 (4.2 - 5.5) |
| Depression (CES-D scale >=3) | 19.2 (17.8 - 20.6) | 18.6 (17.4 - 19.9) |
| ADL >=1 | 15.5 (14.3 - 16.7) | 12.9 (11.8 - 14) |
| IADL >=1 | 15.2 (14.1 - 16.3) | 31.1 (29.5 - 32.6) |
| Often troubled with pain | 43.5 (41.7 - 45.2) | 39.8 (38.2 - 41.4) |
| Ever smoked cigarettes | 62.5 (60.7 - 64.2) | 55 (53.3 - 56.6) |
| Current smoker | 8.8 (7.7 - 9.9) | 12.9 (11.8 - 14.1) |
| Heavy drinking | 27.1 (25.3 - 29) | 11.1 (10.1 - 12.2) |

ADL=Activity of Daily Living; IADL= Instrumental Activity of Daily Living

**Table A2** Comparison of self and clinical health outcomes, in England and the United States, ages 50+, prevalence (95% confidence interval) adjusted for age, sex and BMI.

|  | **England** | **United States** |
| --- | --- | --- |
| *Unweighted-sample N* | 1,946 | 4,575 |
| **Diabetes, HbA1c 6.5** |  |  |
| Prevalence, self-report | 10.5 (8.9 - 12) | 20.2 (18.7 - 21.7) |
| Prevalence, clinical report | 9 (7.5 - 10.5) | 12 (10.8 - 13.1) |
| Prevalence, clinical report corrected for medications | 10.6 (9 - 12.1) | 17.5 (16.1 - 18.9) |
| **Hypertension, systolic blood pressure ≥ 140 mm Hg, diastolic ≥ 90mm Hg,** or taking medication, % |  |  |
| Prevalence, self-report | 37.8 (35.1 - 40.4) | 56.2 (54.2 - 58.1) |
| Prevalence, clinical report | 29.6 (27.3 - 31.9) | 26.5 (24.8 - 28.2) |
| Prevalence, clinical report corrected for medications | 38.4 (35.9 - 40.9) | 39.6 (37.7 - 41.5) |
| C-reactive protein, mg/L |  |  |
| Low risk,<=1, % | 31.3 (29 - 33.7) | 25.5 (23.8 - 27.2) |
| Moderate risk, 1-3, % | 41.8 (39.8 - 43.8) | 41.6 (39.6 - 43.6) |
| High risk, >=3, % | 26.9 (24.6 - 29.1) | 32.8 (31 - 34.7) |
| Mean | 3.3 (3 - 3.7) | 3.6 (3.4 - 3.7) |
| **HDL cholesterol, mg/dL** |  |  |
| Low, 40, % | 11.6 (10.3 - 12.9) | 15.1 (13.8 - 16.4) |
| Normal, 40-60, % | 39.7 (37.7 - 41.7) | 43.8 (41.8 - 45.7) |
| High, 60, % | 48.7 (46.3 - 51.1) | 41.1 (39.2 - 43.1) |
| corrected for medications Low, 40, % | 13.2 (11.8 - 14.6) | 18.8 (17.3 - 20.2) |
| corrected for medications Normal, 40-60, % | 40.1 (38.1 - 42.1) | 44.6 (42.7 - 46.6) |
| corrected for medications High, 60, % | 46.7 (44.2 - 49.1) | 36.6 (34.7 - 38.5) |
| Mean | 61.4 (60.5 - 62.3) | 58.3 (57.6 - 59) |
| Mean corrected for medications | 60.1 (59.3 - 61) | 56.2 (55.6 - 56.9) |
| **Grip strength (kg),** mean | 30.7 (30.3 - 31) | 30.2 (29.9 - 30.4) |
| **Walking speed (m/s)** ^ǂ^**,** mean | 3.4 (3.3 - 3.5) | 3.8 (3.7 - 3.9) |

ǂ unweighted sample size (aged 65+) N=1441 in England, N=2345 in the US

**Figure A2** Distribution of Body Mass Index (BMI) in England and the United States, by age group


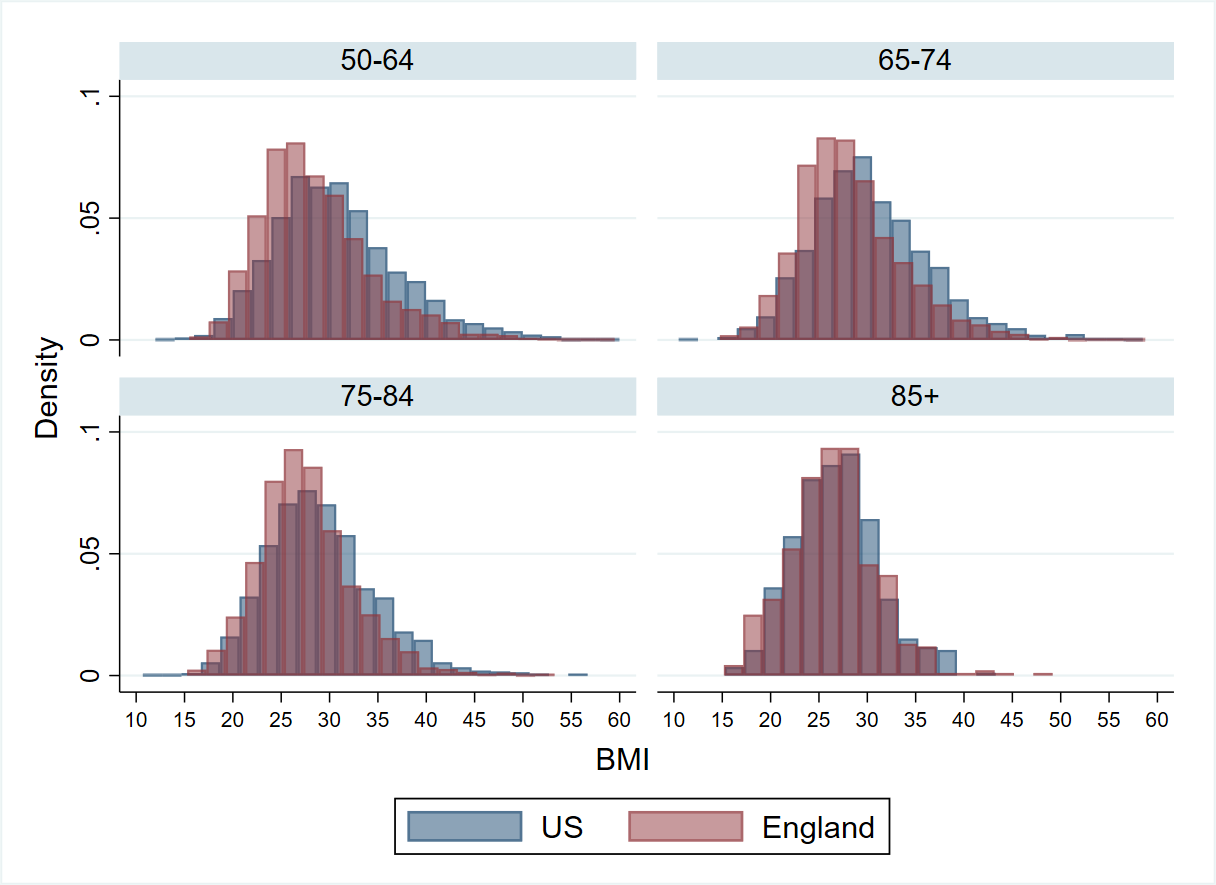


**Table A3** Self-reported health outcomes and health-related behaviors in England and the United States, by age group, prevalence (95% confidence interval).

|  | Aged 50 to 64 | | Aged 65 to 74 | | Aged 75 to 84 | | Aged 85+ | |
| --- | --- | --- | --- | --- | --- | --- | --- | --- |
|  | England | United States | England | United States | England | United States | England | United States |
| Unweighted-sample N | 1,579 | 3,169 | 2,580 | 1,632 | 1,459 | 1,458 | 366 | 424 |
| Hypertension | 27.7 (24.5 - 30.9) | 45.3 (42.9 - 47.8) | 40.1 (38 - 42.2) | 63.5 (60.6 - 66.4) | 54.3 (51.6 - 57) | 70.1 (67.5 - 72.8) | 56.5 (50.9 - 62.2) | 70.4 (65.5 - 75.3) |
| Diabetes | 8.6 (6.8 - 10.4) | 19.2 (17.4 - 21) | 13.6 (12.1 - 15) | 26.7 (24.2 - 29.3) | 16.3 (14.3 - 18.4) | 28.5 (25.9 - 31) | 13.2 (9.1 - 17.3) | 18.7 (14.5 - 22.8) |
| Chronic lung diseases | 3 (1.9 - 4.1) | 8.4 (7 - 9.7) | 6.7 (5.5 - 7.8) | 10.1 (8.3 - 11.8) | 8.1 (6.6 - 9.6) | 14.5 (12.5 - 16.4) | 3.9 (1.9 - 5.9) | 12 (8.3 - 15.6) |
| Cancer | 3.7 (2.4 - 4.9) | 9.9 (8.4 - 11.4) | 6.5 (5.4 - 7.5) | 17.6 (15.4 - 19.9) | 9.7 (8.1 - 11.3) | 24 (21.6 - 26.4) | 6.3 (3.7 - 8.9) | 29.3 (24.6 - 34.1) |
| Stroke | 1.6 (0.9 - -2.3) | 4.1 (3.2 - 4.9) | 4.4 (3.4 - 5.3) | 5.8 (4.4 - 7.2) | 8.3 (6.7 - 9.7) | 9.7 (8.1 - 11.3) | 14.2 (10.2 - 18.1) | 12.9 (9.5 - 16.3) |
| Heart attack | 1.8 (0.9 - -2.5) | 2.8 (1.9 - 3.6) | 4.8 (3.8 - 5.8) | 7.1 (5.6 - 8.5) | 7.6 (6.1 - 9.2) | 12.2 (10.2 - 14.1) | 13.7 (9.5 - 17.7) | 11.7 (8.5 - 14.9) |
| Self-rated general health: Excellent | 12 (9.8 - 14.1) | 9.4 (7.9 - 10.8) | 7.6 (6.1 - 9.1) | 8 (6.3 - 9.6) | 5.2 (4 - 6.3) | 7 (5.6 - 8.5) | 3.9 (2.1 - 5.6) | 5.9 (3.6 - 8.2) |
| Very good | 37.3 (34.1 - 40.5) | 33.2 (30.8 - 35.5) | 35 (32.4 - 37.6) | 35.9 (33 - 38.8) | 23.2 (20.9 - 25.6) | 28.4 (25.8 - 31) | 21.9 (17.3 - 26.4) | 29.1 (24.3 - 34) |
| Good | 31.9 (29.4 - 34.4) | 34.1 (31.8 - 36.4) | 34 (31.2 - 36.7) | 33.6 (30.8 - 36.4) | 38.8 (36 - 41.6) | 38.5 (35.7 - 41.3) | 36.4 (31.4 - 41.5) | 36.8 (31.7 - 42) |
| Fair | 15.2 (13 - 17.4) | 18.6 (16.8 - 20.4) | 18.8 (16.7 - 20.9) | 18.1 (15.9 - 20.3) | 24.1 (21.6 - 26.6) | 19.7 (17.5 - 21.8) | 26.6 (21.4 - 31.7) | 20.6 (16.5 - 24.7) |
| Poor | 3.7 (2.7 - 4.6) | 4.8 (3.8 - 5.8) | 4.6 (3.4 - 5.8) | 4.4 (3.2 - 5.5) | 8.7 (6.9 - 10.4) | 6.4 (5 - 7.8) | 11.2 (7.6 - 14.8) | 7.5 (5 - 10) |
| Depression (CES-D scale >=3) | 19.5 (16.6 - 22.3) | 20.8 (18.9 - 22.8) | 16.5 (14.8 - 18.1) | 15.3 (13.2 - 17.4) | 19.4 (17.2 - 21.5) | 16.1 (14 - 18.2) | 27.5 (22.4 - 32.5) | 20.6 (16.5 - 24.6) |
| ADL >=1 | 10.3 (8.3 - 12.3) | 12.2 (10.7 - 13.7) | 15.6 (14 - 17.3) | 12.2 (10.2 - 14.1) | 20.1 (17.9 - 22.3) | 16.7 (14.6 - 18.8) | 40.3 (34.7 - 45.9) | 26.9 (22.4 - 31.5) |
| IADL >=1 | 11 (9 - 13) | 25 (22.9 - 27.1) | 15.8 (14.1 - 17.4) | 29.6 (26.9 - 32.3) | 24.4 (22 - 26.8) | 49.2 (46.3 - 52.1) | 48.9 (43.2 - 54.5) | 70.2 (65.3 - 75) |
| Often troubled with pain | 38.7 (35.2 - 42.2) | 41.1 (38.7 - 43.5) | 42 (39.9 - 44.1) | 40.2 (37.3 - 43.1) | 43.5 (40.8 - 46.2) | 37.5 (34.7 - 40.2) | 50.1 (44.2 - 55.9) | 33.8 (28.9 - 38.7) |
| Ever smoked cigarettes | 57.8 (54.1 - 61.4) | 53.4 (50.9 - 55.8) | 66.9 (64.9 - 68.9) | 58.5 (55.5 - 61.5) | 64 (61.4 - 66.6) | 56.4 (53.6 - 59.3) | 68.2 (62.8 - 73.7) | 47.7 (42.3 - 53.1) |
| Current smoker | 15.1 (12.3 - 17.8) | 21.8 (19.7 - 23.9) | 9.6 (8.2 - 10.9) | 11.5 (9.6 - 13.4) | 5.4 (4 - 6.8) | 5.3 (3.9 - 6.7) | 2 (0.2 - -3.8) | 3 (1 - 4.9) |
| Heavy drinking | 36.5 (32.8 - 40.1) | 10.9 (9.2 - 12.5) | 29.2 (27.2 - 31.2) | 12.6 (10.6 - 14.6) | 21.9 (19.6 - 24.2) | 12.6 (10.6 - 14.5) | 16.4 (12.4 - 20.3) | 11.1 (7.9 - 14.3) |

SRH=self-rated health; ADL=Activity of Daily Living; IADL= Instrumental Activity of Daily Living

**Table A4** Self-reported and clinical health outcomes in England and the United States, ages 50+, prevalence/mean (95% confidence interval).

|  | **Aged 50 to 64** | | **Aged 65 to 74** | | **Aged 75 to 84** | | **Aged 85+** | |
| --- | --- | --- | --- | --- | --- | --- | --- | --- |
|  | England | United States | England | United States | England | United States | England | United States |
| Unweighted-sample N | 684 | 2,132 | 889 | 1,111 | 684 | 2,132 | 889 | 1,111 |
| **Diabetes**, HbA1c >=6.5 |  |  |  |  |  |  |  |  |
| Self-report | 7.1 (4.9 - 9.3) | 18.3 (16.2 - 20.3) | 11.5 (9 - 13.8) | 25.1 (22.1 - 28.1) | 14.6 (10.9 - 18.3) | 26 (23.2 - 28.8) | 10.6 (4.4 - 16.7) | 15.7 (11.1 - 20.2) |
| Clinical report | 5.7 (3.6 - 7.7) | 11.2 (9.5 - 12.8) | 10.1 (7.8 - 12.4) | 15.6 (13.1 - 18.2) | 13.9 (9.8 - 18.1) | 16.5 (14.1 - 18.8) | 7.6 (2.2 - 12.9) | 9 (5.3 - 12.6) |
| Clinical report corrected for medications | 6.1 (4.1 - 8.1) | 15.6 (13.7 - 17.6) | 12 (9.5 - 14.5) | 22.8 (19.9 - 25.8) | 17.4 (12.9 - 21.8) | 23.6 (20.8 - 26.3) | 10.2 (4.2 - 16.1) | 14.3 (9.9 - 18.7) |
| **Hypertension**, systolic blood pressure 140 mm Hg, diastolic 90mm Hg, or taking medication, % |  |  |  |  |  |  |  |  |
| Self-report | 25.5 (21.4 - 29.5) | 45.7 (42.9 - 48.6) | 40 (36.4 - 43.7) | 62 (58.5 - 65.6) | 53.1 (48.2 - 58) | 69.9 (66.8 - 73) | 51.3 (41.4 - 61.2) | 72 (66.2 - 77.9) |
| Clinical report | 22.4 (18.6 - 26.2) | 24.9 (22.4 - 27.4) | 31.8 (28.4 - 35.3) | 27.3 (24 - 30.5) | 36 (31.3 - 40.7) | 30 (27 - 33) | 38.2 (28.4 - 48) | 34 (28 - 40) |
| Clinical report corrected for medications | 27.4 (23.4 - 31.3) | 36.8 (34 - 39.6) | 41.1 (37.5 - 44.7) | 41.6 (38.1 - 45.2) | 47.5 (42.6 - 52.4) | 44.8 (41.5 - 48.1) | 47.2 (37.2 - 57.3) | 47 (40.6 - 53.4) |
| **C-reactive protein**, mg/L |  |  |  |  |  |  |  |  |
| Low risk,<=1, % | 40.3 (35.9 - 44.6) | 26.4 (23.7 - 29) | 35.8 (32.3 - 39.3) | 27.3 (24.1 - 30.6) | 33.4 (28.9 - 37.8) | 29.2 (26.1 - 32.2) | 31.7 (22.4 - 41) | 33.6 (27.6 - 39.6) |
| Moderate risk, 1-3, % | 36.1 (33.2 - 38.9) | 36.8 (34 - 39.6) | 38.8 (35.2 - 42.3) | 39.1 (35.5 - 42.6) | 40.8 (37.6 - 44.1) | 41.1 (37.9 - 44.4) | 36.4 (30.3 - 42.5) | 36.4 (30.3 - 42.4) |
| High risk, >=3, % | 23.7 (20.2 - 27.2) | 36.9 (34.1 - 39.7) | 25.4 (22.2 - 28.6) | 33.6 (30.2 - 37) | 25.8 (21.8 - 29.8) | 29.7 (26.7 - 32.7) | 31.9 (22.6 - 41.3) | 30.1 (24.1 - 36.1) |
| Mean | 2.7 (2.3 - 3.1) | 3.8 (3.5 - 4) | 3.1 (2.6 - 3.7) | 3.4 (3.1 - 3.7) | 3.4 (2.4 - 4.4) | 3.2 (2.9 - 3.5) | 4.3 (2.4 - 6.2) | 3.3 (2.7 - 3.9) |
| **HDL cholesterol**, mg/dL |  |  |  |  |  |  |  |  |
| Low, ≤40 | 10.7 (8.6 - 12.8) | 13.9 (12 - 15.8) | 12.1 (9.9 - 14.2) | 16.4 (13.7 - 19) | 11.5 (9.1 - 13.9) | 19.8 (17.2 - 22.5) | 9.2 (4.8 - 13.6) | 14.6 (10.5 - 18.7) |
| Normal, 40-60 | 39.4 (36 - 42.8) | 43.6 (40.6 - 46.5) | 36.7 (33.5 - 39.9) | 41.2 (37.6 - 44.8) | 36.4 (32.8 - 40) | 43.7 (40.4 - 47.1) | 43.2 (34.9 - 51.5) | 50.4 (44.1 - 56.8) |
| High, ≥60 | 49.9 (45.5 - 54.3) | 42.6 (39.7 - 45.5) | 51.2 (47.8 - 54.6) | 42.5 (38.9 - 46.1) | 52.1 (47.3 - 56.9) | 36.4 (33.2 - 39.6) | 47.6 (36.6 - 58.5) | 35 (28.9 - 41.1) |
| corrected for medications Low, ≤40 | 12 (9.7 - 14.3) | 16.7 (14.6 - 18.8) | 13.4 (11.1 - 15.7) | 20 (17.1 - 22.8) | 14.6 (11.9 - 17.3) | 26.7 (23.8 - 29.7) | 11.3 (6.1 - 16.5) | 21.2 (16.2 - 26.2) |
| corrected for medications Normal, 40-60 | 39.2 (35.9 - 42.6) | 44 (41.1 - 46.9) | 38.2 (35 - 41.5) | 43.2 (39.6 - 46.9) | 36.2 (32.8 - 39.5) | 42 (38.7 - 45.4) | 42.2 (34.3 - 50.1) | 49.6 (43.2 - 56) |
| corrected for medications High, ≥60 | 48.8 (44.2 - 53.3) | 39.3 (36.5 - 42.2) | 48.3 (44.8 - 51.9) | 36.8 (33.2 - 40.4) | 49.3 (44.7 - 53.9) | 31.2 (28.2 - 34.3) | 46.5 (35.5 - 57.5) | 29.2 (23.4 - 35) |
| *Mean* | 62.1 (60.5 - 63.7) | 59.2 (58.2 - 60.2) | 62.3 (61 - 63.6) | 58.5 (57.1 - 59.8) | 62.6 (60.8 - 64.4) | 56.1 (54.9 - 57.3) | 61.3 (57.7 - 64.9) | 55.2 (53.3 - 57.1) |
| *Mean corrected for medications* | 61.4 (59.7 - 63) | 57.6 (56.6 - 58.6) | 60.8 (59.5 - 62.1) | 56 (54.7 - 57.4) | 60.8 (59.1 - 62.6) | 53.2 (51.9 - 54.4) | 59.7 (56 - 63.5) | 52.9 (50.9 - 54.8) |
| **BMI** |  |  |  |  |  |  |  |  |
| Normal weight | 28.2 (24.5 - 31.9) | 18.5 (16.1 - 20.8) | 26.1 (22.9 - 29.4) | 16.3 (13.7 - 18.9) | 33.1 (29 - 37.3) | 24.3 (21.3 - 27.2) | 33.7 (24.2 - 43.2) | 33.9 (27.9 - 39.9) |
| Overweight | 36.1 (33.1 - 39.2) | 32.5 (29.8 - 35.2) | 41.4 (37.5 - 45.3) | 37.1 (33.6 - 40.6) | 39.9 (36.5 - 43.2) | 39.3 (36.1 - 42.6) | 48.6 (41.8 - 55.5) | 48.5 (42.2 - 54.9) |
| Class I Obese | 21.9 (19.3 - 24.4) | 27.3 (24.7 - 29.9) | 21.4 (18.7 - 24.1) | 28.2 (24.9 - 31.4) | 18.1 (15.4 - 20.9) | 23.3 (20.5 - 26.1) | 11.2 (6.6 - 15.8) | 11.1 (7.4 - 14.8) |
| Class II obese | 9.1 (7.3 - 10.8) | 13.9 (11.9 - 15.8) | 8.1 (6.4 - 9.8) | 13.3 (10.8 - 15.7) | 6.9 (5.2 - 8.5) | 10.1 (8.1 - 12) | 6.2 (2.5 - 9.9) | 6.1 (2.9 - 9.2) |
| Class IIIobese | 4.7 (3.5 - 5.9) | 7.9 (6.3 - 9.5) | 2.9 (1.9 - 3.9) | 5.2 (3.6 - 6.8) | 2 (1.1 - 2.8) | 3 (1.9 - 4.1) | 0.3 (-0.3 - 1) | 0.3 (-0.3 - 0.9) |
| *Mean* | 28.6 (28.1 - 29.1) | 30.5 (30.2 - 30.9) | 28.4 (28 - 28.8) | 30.3 (29.8 - 30.7) | 27.6 (27.1 - 28) | 28.9 (28.5 - 29.2) | 26.8 (25.9 - 27.6) | 26.9 (26.3 - 27.4) |
| **Greep strength (kg)**: mean | 33.4 (32.8 - 34.1) | 33.1 (32.7 - 33.5) | 30 (29.5 - 30.5) | 29.7 (29.2 - 30.3) | 25.4 (24.8 - 26) | 25.2 (24.8 - 25.6) | 19.7 (18.6 - 20.8) | 19.5 (18.8 - 20.2) |
| **Walking speed (m/s)** ^ǂ^: mean |  |  | 3 (2.8 - 3.1) | 3.3 (3.3 - 3.4) | 3.5 (3.3 - 3.7) | 4 (3.8 - 4.2) | 4.8 (4.1 - 5.5) | 5.1 (4.9 - 5.4) |

BMI=body mass index; Class I obesity: BMI 30-35; Class II obesity: BMI 35-39; Class III obesity: BMI >=40

^ǂ^ aged 65+

ǂ unweighted sample size (aged 65+) N=1441 in England, N=2345 in the US

**Figure A3: Country differences in health outcomes**

1. **Self-reported outcomes**

**
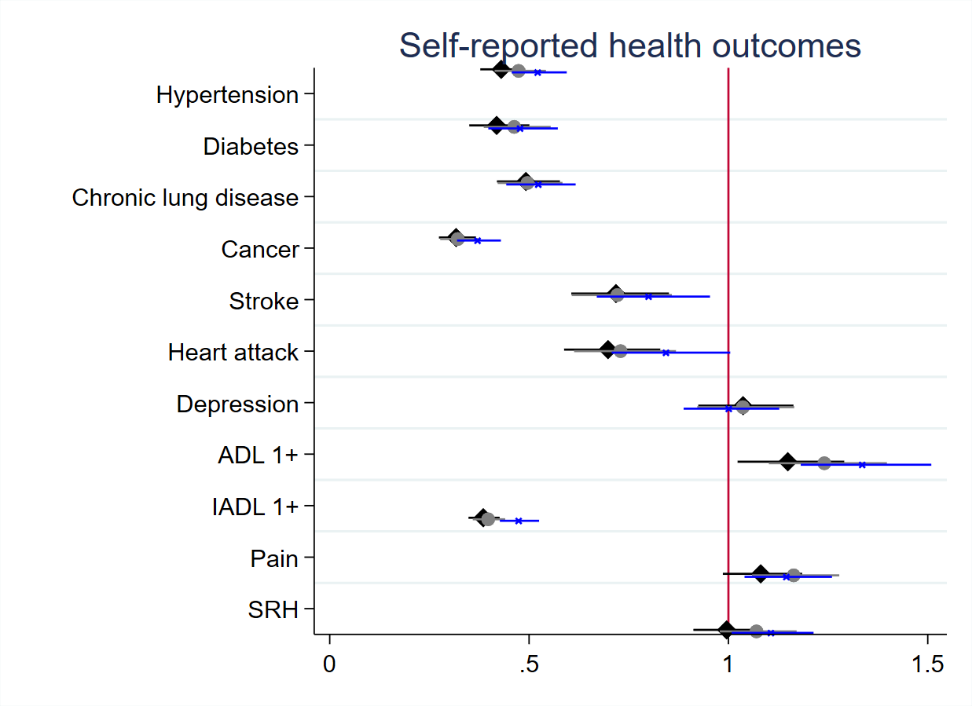
**

**♦ England vs US odds ratio of logistic and ordered logistic (SRH) model adjusted by sex and age**

**Χ England vs US odds ratio of logistic and ordered logistic (SRH) model adjusted by sex and BMI**

**• England vs US odds ratio of logistic and ordered logistic (SRH) model adjusted by sex, age and BMI**

1. **Biomarker outcomes**


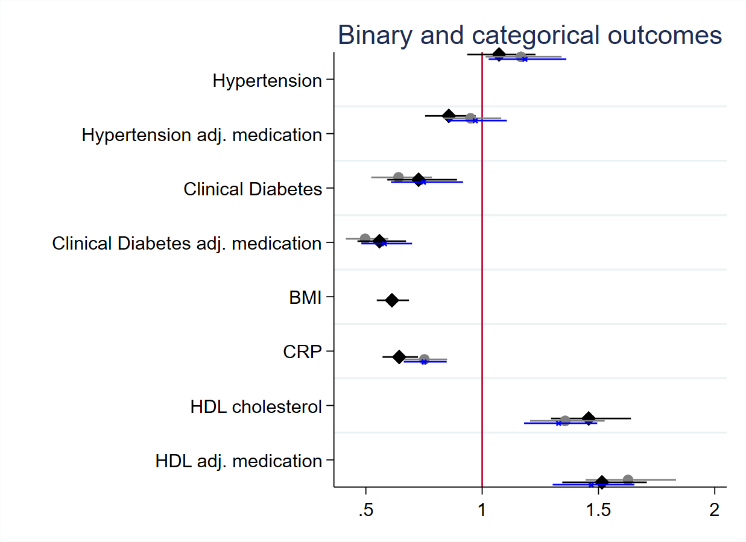
**
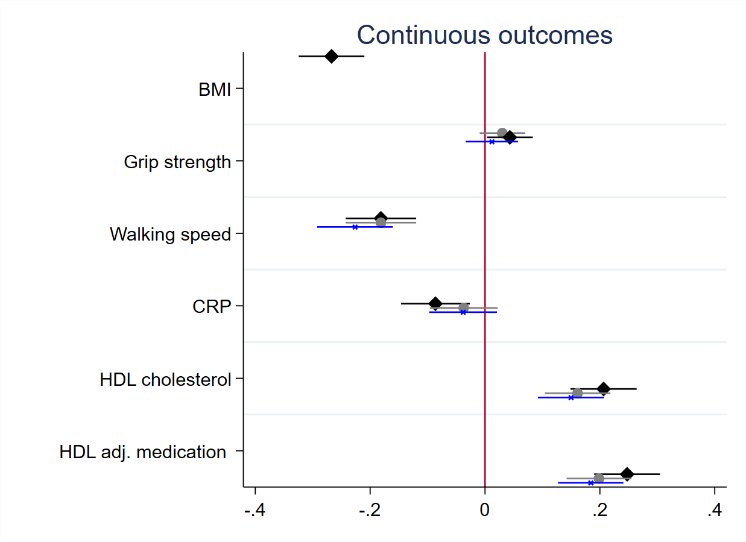
**

**♦ England vs US odds ratio of logistic and ordered logistic (BMI, CRP, HDL) model adjusted by sex and age**

**Χ England vs US odds ratio of logistic and ordered logistic (BMI, CRP, HDL) model adjusted by sex and BMI**

**• England vs US odds ratio of logistic and ordered logistic (BMI, CRP, HDL) model adjusted by sex, age and BMI**

**♦ England vs US coefficient of OLS model adjusted by sex and age**

**Χ England vs US coefficient of OLS model adjusted by sex and BMI**

**• England vs US coefficient of OLS adjusted by sex, age and BMI**

*Note:* Categories of **HDL cholesterol** are coded “Low”, <40%, “normal” 40-60%, “high” 60%, “High HDL” corresponds to healthier condition, similarly for continuous variable higher values correspond to better health status.

For **walking speed** higher values correspond to poorer health status.

For **grip strength** higher values correspond to better health status.

**Figure A4: Country differences in health outcomes by age group**

1. **Self-reported outcomes**

**
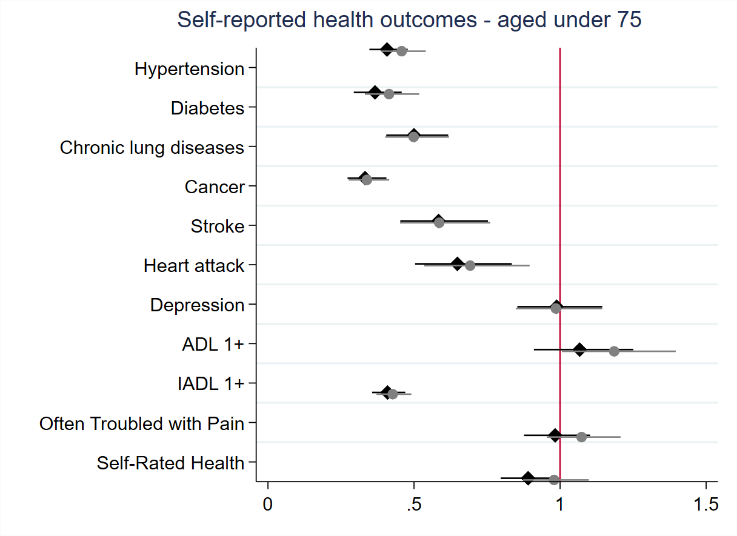

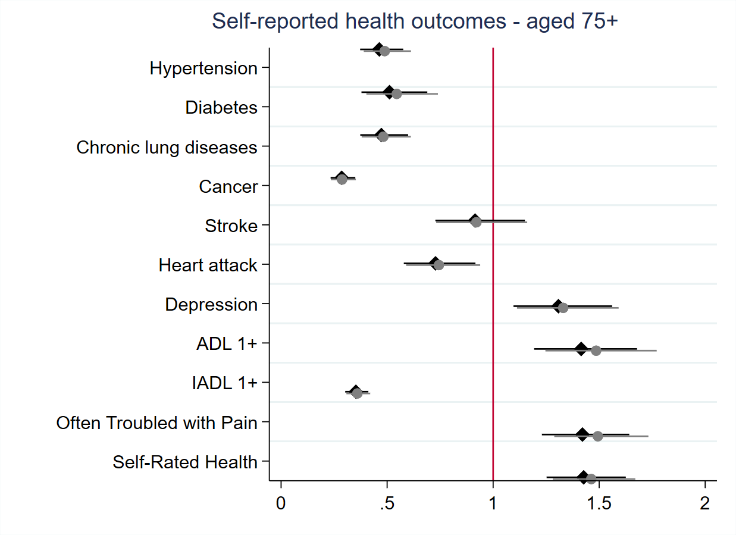
**

1. **Biomarker outcomes – categorical**


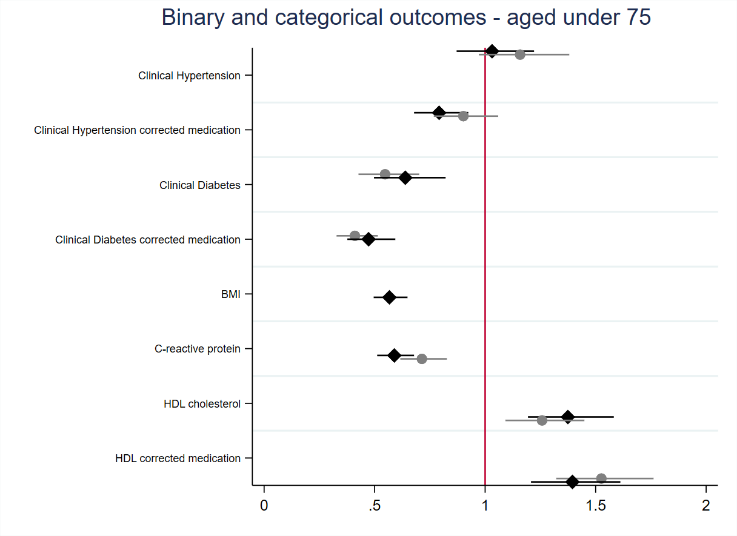

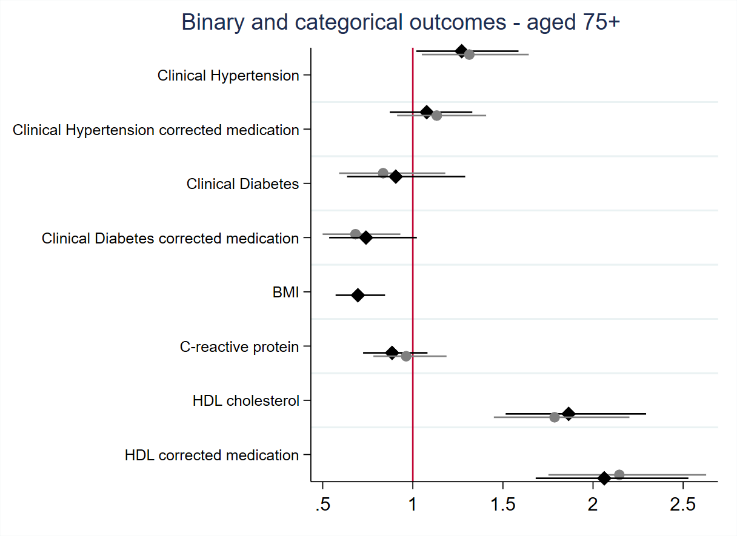


1. **Biomarker outcomes – continuous**


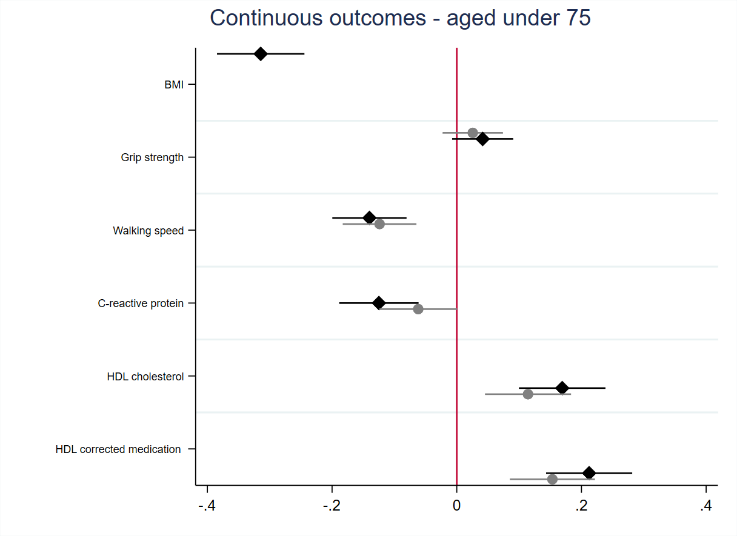

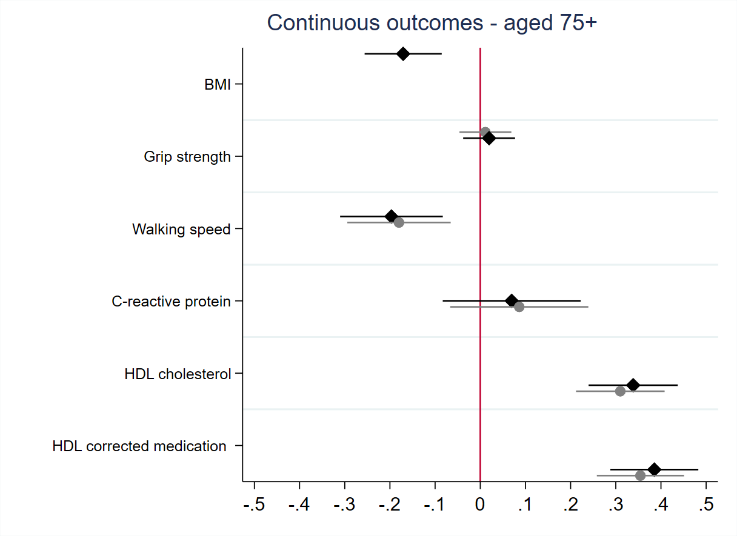


**♦ England vs US odds ratio of logistic and ordered logistic and England vs US coefficients of OLS models adjusted by sex and age**

**• England vs US odds ratio of logistic and ordered logistic and England vs US coefficients of OLS models adjusted by sex, age and BMI**

*Note:* Categories of **HDL cholesterol** are coded “Low”, <40%, “normal” 40-60%, “high” 60%, “High HDL” corresponds to healthier condition, similarly for continuous variable higher values correspond to better health status.

For **walking speed** higher values correspond to poorer health status.

For **grip strength** higher values correspond to better health status.

**Figure A5** Proportion of Covid-19 deaths on all-cause deaths by age group, for all deceases registered in 2020


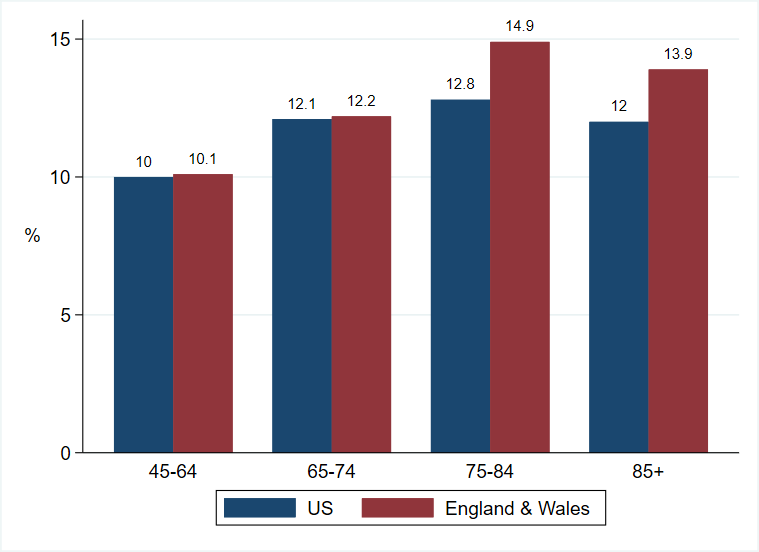

Supplement: gbac023_suppl_Supplementary_Appendix [file gbac023_suppl_supplementary_appendix.docx]
